# Economic cost analysis of malaria case management at the household level during the malaria elimination phase in The People’s Republic of China

**DOI:** 10.1186/s40249-016-0141-x

**Published:** 2016-06-03

**Authors:** Shang Xia, Jin-Xiang Ma, Duo-Quan Wang, Shi-Zhu Li, David Rollinson, Shui-Sen Zhou, Xiao-Nong Zhou

**Affiliations:** National Institute of Parasitic Diseases, Chinese Center for Disease Control and Prevention, Shanghai, 200025 People’s Republic of China; Key Laboratory of Parasite and Vector Biology, Ministry of Health, Shanghai, 200025 People’s Republic of China; WHO Collaborating Center for Tropical Diseases, Shanghai, 200025 People’s Republic of China; Department of Applied Statistics, School of Public Health, Guangzhou Medical University, Guangzhou, 510182 China; Life Sciences Department, The Natural History Museum, Cromwell Road, London, SW7 5BD UK

**Keywords:** Malaria, Economic cost analysis, Direct costs, Indirect costs, Health facilities, China

## Abstract

**Background:**

In China, malaria has been posing a significant economic burden on households. To evaluate malaria economic burden in terms of both direct and indirect costs has its meaning in improving the effectiveness of malaria elimination program in China.

**Methods:**

A number of study sites (eight counties in five provinces) were selected from the malaria endemic area in China, representing the different levels of malaria incidence, risk classification, economic development. A number of households with malaria cases (*n* = 923) were surveyed during the May to December in 2012 to collect information on malaria economic burden. Descriptive statistics were used to characterize the basic profiles of selected malaria cases in terms of their gender, age group, occupation and malaria type. The malaria economic costs were evaluated by direct and indirect costs. Comparisons were carried out by using the chi-square test (or Z-test) and the Mann-Whitney U test among malaria cases with reference to local/imported malaria patients, hospitalized/out patients, and treatment hospitals.

**Results:**

The average cost of malaria per case was 1 691.23 CNY (direct cost was 735.41 CNY and indirect cost was 955.82 CNY), which accounted for 11.1 % of a household’s total income. The average costs per case for local and imported malaria were 1 087.58 CNY and 4271.93 CNY, respectively. The average cost of a malaria patient being diagnosed and treated in a hospital at the county level or above (3 975.43 CNY) was 4.23 times higher than that of malaria patient being diagnosed and treated at a village or township hospital (938.80 CNY).

**Conclusion:**

This study found that malaria has been posing a significant economic burden on households in terms of direct and indirect costs. There is a need to improve the effectiveness of interventions in order to reduce the impact costs of malaria, especially of imported infections, in order to eliminate the disease in China.

**Electronic supplementary material:**

The online version of this article (doi:10.1186/s40249-016-0141-x) contains supplementary material, which is available to authorized users.

## Multilingual abstract

Please see Additional file 1 for translations of the abstract into the six official working languages of the United Nations.

## Background

Malaria has long been known as a disease of poverty as it is mainly distributed in the poorest regions of the world, e.g., in African countries [1, 2]. Existing studies have demonstrated the striking correlations that exist between malaria and poverty in terms of the in terms of a country’s GDP per capita; the studies have shown that malaria-endemic countries are characterized by a relatively lower level of economic development. In this regard, malaria is beyond a major health problem: it also has significance on the socioeconomic development of poor countries [3, 4]. Furthermore, the relationship between malaria and poverty demonstrates the positive correlations: malaria infections may result in poverty on both the individual and national level and, in turn, poverty leads to worsened health conditions that may aggravate malaria transmission [4, 5]. Malaria accounts for a loss of 35 million disability-adjusted life years annually [6, 7]. Besides the economic loss caused by malaria, the disease can also cause indirect loss in the national level economic development, such as the reductions on the foreign direct investment, tourism, labor productivity, and international trade. At the individual level, malaria may cause poverty due to expenditures on healthcare and treatments, income losses, and early deaths. People living in poverty are considered to be a high-risk population, as they are less likely to be able to access preventive measures or seek prompt effective treatment [4, 8–10].

Given the remarkable success that China has already achieved, and the confidence that it can achieve even more, a strong political commitment was made by the National Population and Family Planning Commission of the Peoples’ Republic of China by the issuing of the National Action Plan for Malaria Elimination (2010–2020). The goal is to eliminate locally acquired malaria by the end of 2015, except for in the border areas of Yunnan Province, and to eliminate malaria nationwide by 2020 [11]. With the continuous reduction of locally acquired malaria, the landscape of malaria endemics have changed significantly, which generates a new set of challenges [12]. One of these is the changing proportions of local and imported cases [13].

The proportion of imported malaria cases has increased significantly due to the growing overseas investment, and the numbers of Chinese laborers working abroad and then returning home. In this analysis, an imported case of malaria was defined as case of malaria acquired in a known malarious area outside of China. In China, the following criteria for imported malaria must be simultaneously met: 1) the patient was given a diagnosis of malaria; 2) the patient has a history of travel to malaria-endemic areas outside of China during the malaria transmission season; and 3) the onset time for malaria was <1 month after returning to China during the local transmission season.

The total number of Chinese laborers and travelers abroad in 2012 was estimated to be 0.5 million and 83.2 million, respectively, which increased by 24.6 and 44.9 %, respectively, when comparing with figures from 2010 [14, 15]. According to a web-based surveillance and reporting system, although only a total of 2 570 malaria cases were reported in 2012, a reduction of 67.28 % compared with the 7 855 cases reported in 2010, the proportion of imported cases increased sharply from 54.26 % (4 262/7 855) in 2010 to 94.71 % (2 434/2 570) in 2012.

Although malaria still poses a health and economic burden for the poor population in China and imported malaria remains a potential threat [16], only a limited number of studies have been conducted on malaria management costs at the household level. There are only two related available reports focusing on cost-effectiveness of malaria programs in China [17, 18]. The economic cost of endemic malaria has received little attention and has not been adequately documented until now. Information on the economic consequences of getting infected with malaria at the household level is an important complementary tool needed to successfully formulate policies in areas such as health financing, introduce innovative strategies, and regulate health facilities [19–21].

This is the first study based on a systematic survey conducted in the main malaria endemic areas that endeavors to give an in-depth analysis of malaria management costs at the household level throughout the country. Having this information could help prioritize current interventions for malaria elimination.

## Methods

### Study sites

**Fig. 1:**
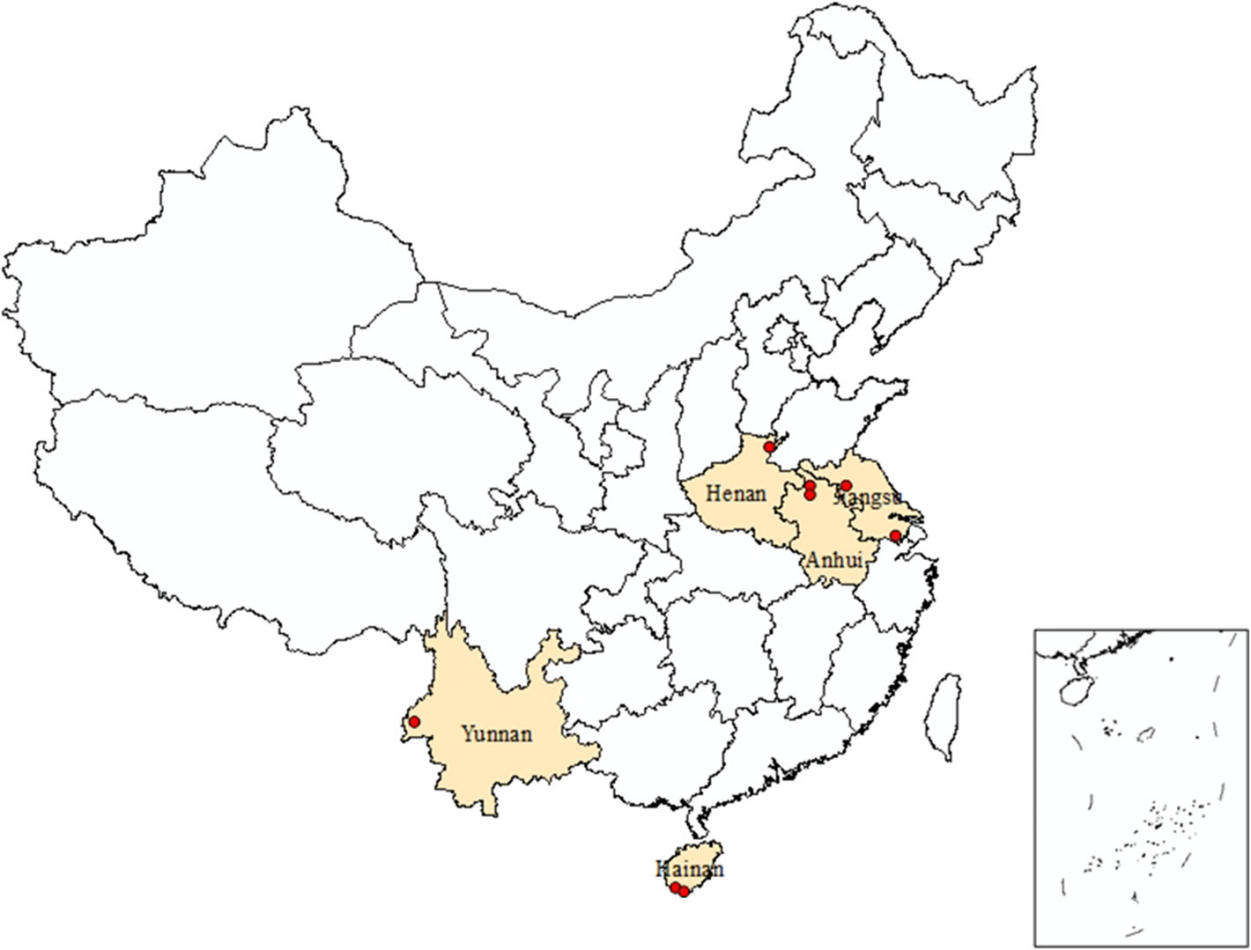
The eight study sites (red dots) selected from five provinces in China

In order to represent the different situations of malaria endemic area in China, we took into consideration of the different levels of malaria incidence, risk classification, economic development and selected eight sample counties in five provinces, including Jiangsu, Anhui, Henan, Yunnan, and Hainan. The first four counties, i.e., Sihong in Jiangsu, Guoyang in Anhui, Yongcheng in Henan, and Sanya in Hainan were selected to examine the cost of local malaria, while the another four counties, i.e., Suzhou in Jiangsu, Puyang in Henan, Tengchong in Yunnan, and Ledong in Hainan were selected to study the cost of imported malaria (see Fig. 1).

### Sampling and data collection

We collected information on reported malaria cases in each selected county, based on surveillance data from the Chinese Information System for Disease Control and Prevention. All cases were laboratory confirmed by microscopy and positive cases were treated according to national policy. A questionnaire was developed based on a workshop presented by stakeholders in order to collect the following data: background information, healthcare-seeking behavior, working days lost, and expenditure on malaria diagnosis and treatment.

From May to December 2012, all selected malaria cases (*n* = 923) were interviewed using the standard questionnaire. A principal supervisor and two investigators monitored the data collection process. A household was defined as a person with his spouse, unmarried children, and related or unrelated persons, who live together and constitute one unit. The household head or a representative (i.e., in the situation that household head was not accessible during the interview period) was interviewed to obtain information on treatment-seeking behavior and the amount of money spent to treat malaria. For children subjects, either their mother or both parents were interviewed.

### Data analysis

Economic costs were calculated in Chinese Yuan Renminbi (CNY). Descriptive statistics were used to characterize the basic profiles of selected malaria cases in terms of their gender, age group, occupation and malaria type. The malaria economic costs were evaluated by direct and indirect costs. Comparisons were carried out by using the chi-square test (or Z-test) and the Mann-Whitney U test among malaria cases with reference to local/imported malaria patients, hospitalized/out patients, and treatment hospitals. A *P*-value less than 0.05 was considered statistically significant.

### Classifying costs

Economists classify costs according to their traceability (direct and indirect) to the object or activity [9], or by their relationship to the output (fixed and variable). Direct and indirect costs are used by health economists to classify (demand-side) patient costs associated with illness [11]. In our study, direct costs for all malaria patients included payment for examinations, drugs, and treatment. Indirect costs included the patients’ and caregivers’ lost labor productivity or loss of incomes, as well as costs incurred by caregivers, such as for transportation and food. Productive work was broadly defined as involvement in any economic activity with the potential to add to the disposable income of the household. The total numbers of working days lost by both the patients and their caregivers were recorded. Indirect costs were calculated both at the household level and for cured malaria cases.

### Ethical considerations

We obtained ethics approval from the ethical committee of the National Institute of Parasitic Diseases, Chinese Center for Disease Control and Prevention (WHO Collaborating Center for Malaria, Schistosomiasis and Filariasis). Written informed consent was obtained from all participants. No specific local permissions were required, and the field studies did not involve endangered or protected species.

## Results

As shown in Table 1, the ratio of *P. vivax* (*P. v*) to *P. falciparum* (*P. f*) malaria in the study sample was not dissimilar (Z = 3.514/*P* = 0.061) to the ratio in the entire country. However, the ratio of local to imported cases in our study sample was statistically different (Z = 53.840/*P* < 0.05) compared with the entire country (see Table 1). A total of 923 malaria cases were investigated, 6 of which were removed from the data analysis due to the incorrect responses (*n* = 917). As illustrated in Table 2, most of the study subjects were male (66.1 %), of working age (73 %, 18–60 years old), farmers (69.7 %), and infected with *P. v* (80.7 %) malaria (see Table 2).

**Table 1:** Types of malaria cases (species and local vs. imported) in the study sample compared with the entire country in 2009–2011

Malaria type	Entire country	Study sample	Statistics/*p*-value
*P. v* vs. *P. f*	19,411/3621	740/114	Z = 3.514/*p* = 0.061
Local vs. imported	16,383/6789	751/166	Z = 53.840/*p* < 0.05

**Table 2:** Sociodemographic characteristics of the selected malaria patients

Variable	Number	Proportion (%)
Gender		
Male	606	66.1
Female	311	33.9
Age group		
0~	75	8.21
18~	176	19.26
30~	176	19.26
40~	207	22.65
50~	110	12.04
60~	170	18.6
Missing data	3	
Occupation		
Student	106	11.6
Farmer	635	69.74
Businessman	107	11.71
Others	66	7.22
Missing data	3	
Malaria species		
*P. v*	740	80.70
*P. f*	114	12.43
Other	61	6.65
Missing data	2	0.22

The average cost of malaria per case was 1 691.23 CNY (735.41 CNY for direct costs and 955.82 CNY for indirect costs). As shown in Table 3, the average cost for each local patient was estimated as 1 087.58 CNY, with the average indirect cost almost two times that of the direct cost, i.e., 701.24/372.87. The average cost of an imported malaria case was about 4 times that of a local malaria case, i.e., 4 271.93/1 087.58, and the average indirect cost for an imported case was a little higher than the direct cost for an imported cases, i.e., 2 254.96/2 057.38. For the local cases, the costs of absence from work comprised about half of the total indirect costs, while drug related costs accounted for about 61 % of the direct costs. For the imported cases, the costs of absence from work were about 60 % of the indirect costs, while 45 % of the direct costs were due to drugs.

**Table 3:** Comparison of the direct and indirect costs related to local and imported malaria cases in the study areas

	Local cases		Imported cases		*p*-value
	Mean	SD	Mean	SD	
Direct costs					
Drugs	227.70	65.23	1012.12	29.87	<0.05
Examinations	59.21	19.75	865.16	30.39	<0.05
Treatment	43.45	12.81	562.58	14.57	<0.05
Other	12.67	4.79	172.71	9.36	0.615
Subtotal direct costs	372.87	90.22	2254.96	68.07	<0.05
Indirect costs					
Nutritional supplements	182.65	19.44	277.99	34.48	<0.05
Transportation	43.97	9.10	126.24	18.88	<0.05
Accommodation	95.60	18.59	383.63	38.20	<0.05
Absence of work	352.42	56.26	1233.82	142.97	<0.05
Other	26.60	11.16	35.70	18.46	0.917
Subtotal indirect costs	701.24	82.79	2057.38	207.46	<0.05
Total costs	1087.58	152.15	4271.93	780.13	<0.05

As shown in Table 4, 81.9 % of the cases were outpatients, with the total average cost of 982.39 CNY per case; 71.8 % of this cost was indirect (705.33 CNY), while the direct cost averaged 287.71 CNY. The total average cost of a hospitalized patient was 4.7 times higher than that of an outpatient, i.e., 4 624.62/982.39, and the indirect and direct costs of hospitalized patients were very similar (2 032.17 vs. 2 587.91). The indirect and direct costs of hospitalized patients were 2.9 times and 9 times higher than those of outpatients, respectively.

**Table 4:** Comparison of the direct and indirect costs of hospitalized patients and outpatients in the study areas

	Hospitalized patients		Outpatients		*p*-value
	Mean	SD	Mean	SD	
Direct costs					
Drugs	1169.69	280.48	164.55	42.46	<0.05
Examinations	748.68	269.23	48.3	20.52	<0.05
Treatment	489.02	129.57	35.12	10.93	<0.05
Other	140.32	82.87	171.05	16.79	0.196
Subtotal direct costs	2587.91	136.12	287.71	65.19	<0.05
Indirect costs					
Nutritional supplements	324.3	38.28	171.05	16.79	<0.05
Transportation	136.74	20.19	41.35	8.10	<0.05
Accommodation	385.05	33.81	94.96	20.43	<0.05
Absence of work	1148.23	136.12	371.87	62.77	<0.05
Other	38.76	18.51	26.1	11.11	0.507
Subtotal indirect costs	2032.17	196.78	705.33	89.39	<0.05
Total costs	4624.62	770.59	982.39	128.59	<0.05

**Fig. 2:**
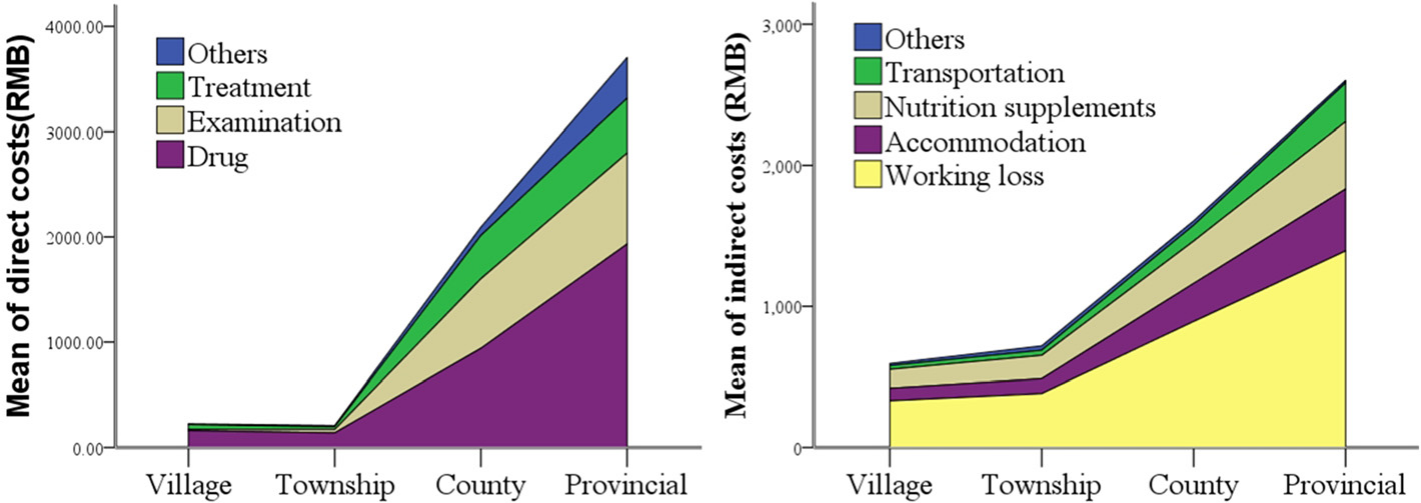
Comparison of the direct and indirect costs incurred at different levels of hospitals in the study areas

As shown in Fig. 2, about 77.3 % of the surveyed malaria patients were diagnosed and treated at a village and/or township hospital, with the average cost of 938.80 CNY. The rest were diagnosed and treated in a hospital at the county level or above, with the average cost of 3 975.43 CNY, which is about 4.23 times higher than for those diagnosed and treated at a village and/or township hospital. The ratio of the average direct cost at a village and/or township hospital to the average direct cost at a county or above hospital was about 1:10 (240.66/2 275.95), and the ratio of the average indirect cost was about 2:5 (702.17/1 724.70).

As shown in Table 5, 19.5 % of the total cases were diagnosed at a village hospital, with 24.6, 51.4, and 24.0 % of these cases receiving treatment at a village, township, and county or above hospital, respectively. Of the total cases, 60.0 % were diagnosed at a township hospital, with 5.8, 90.0, and 4.3 % of these receiving treatments at a village, township, and county or above hospital, respectively. Of the total cases, 14.5 % were diagnosed at a county or above hospital, with 1.5, 22.3, and 76.2 % of these receiving treatments at a village, township and county or above hospital, respectively.

**Table 5:** The distribution of hospitals where malaria patients were diagnosed and treated

Diagnosis hospital	Treatment hospital			Total
	Village	Township	County or above	
Village	43(24.6 %)	90(51.4 %)	42(24.0 %)	175(19.5 %)
Township	31(5.8 %)	484(90.0 %)	23(4.3 %)	538(60.0 %)
County or above	2(1.5 %)	29(22.3 %)	99(76.2 %)	130(14.5 %)
Other^a^	6(11.1 %)	8(14.8 %)	40(74.1 %)	54(6.0 %)
Total	82(9.1 %)	611(68.1 %)	204(22.7 %)	897(100 %)^b^

^a^Including private clinics and others

^b^Another 20 cases had no data about their hospital for treatment

**Fig. 3:**
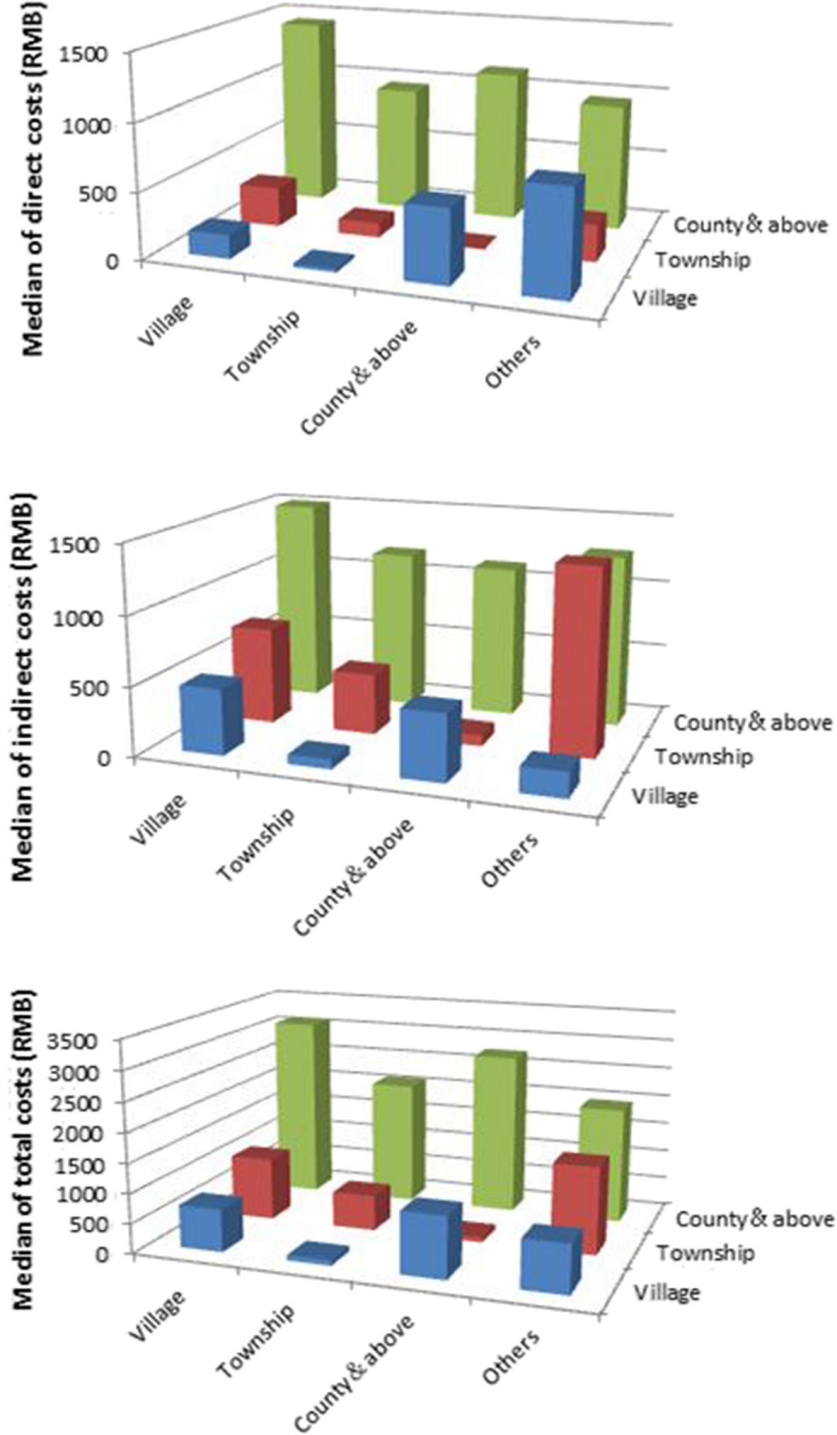
The median of direct, indirect, and total costs incurred at different levels of hospitals (for diagnosis and treatment) in the study areas

As shown in Fig. 3, the cases treated at a hospital at the county level or above incurred relatively higher costs, no matter at which hospital the cases were diagnosed. These observations indicate that malaria patients would be more inclined to be treated at a village or township hospital aiming to save money, although many of them were diagnosed at a county or above hospital with a better medical condition.

## Discussion

### Malaria has been posing a significant economic burden on households in the study areas

This study was the first to analyze direct and indirect costs associated with malaria management during the malaria elimination stage in China. The study found that the average costs for local and imported malaria cases were 1 087.58CNY and 4 271.93CNY, respectively. Although the basic antimalarial drugs have being offered for free in the National Malaria Elimination Programme, many households with malaria patients in the study areas spent their money on more public health services, such as blood examination, and private sources of treatment. The surveyed households spent about 13.9 times more on malaria treatment than the average per capita daily income per year, which accounted for about 11.1 % of a household’s total income. According to studies, the economic losses incurred due to the disease are equivalent to as much as 0.6–1.3 % of the GDP in countries with high malaria incidence rates [22]. With the increasing overseas investment and growing number of Chinese people working abroad, the number of imported malaria cases has also increased sharply, and the costs of imported cases are an unavoidable challenge for the elimination of malaria in China. However, the findings of this study were consistent with those of other studies about household costs of malaria management. This study found that the direct costs were lower than the indirect costs, showing that most of the costs went on drugs and were incurred due to loss of working days in the study areas. Other studies have found that indirect costs constitute more than 75 % of the total costs [23–25]. In Ghana and Sri Lanka, the indirect costs for malaria management accounted for 79 % [24] and 76 % [26] of the total costs, respectively. This study as well as others has shown that the highest proportion of malaria treatment expenditure goes on drugs [3, 4].

### The most striking finding of this study was the pronounced increase in the costs incurred by malaria patients who were treated in a hospital at the county level or above

In terms of the average cost, it was about 4.23 times higher than for those treated at a village or township hospital (i.e., 3 975.43/ 938.80). In terms of the direct cost, it was near 10 times higher for getting treated at a hospital at the county level or above than getting treated at a village or township hospital (i.e., 2 275.95/240.66). Furthermore, 24.0 % of patients who were diagnosed at a village hospital opted to get treated at a county or above hospital, which significantly increases patients’ costs. Therefore, the results confirmed that early diagnosis and quick treatment in the local village or township hospitals would reduce the related costs significantly. Besides the direct and/or indirect medical treatment costs, malaria can impose other types of economic costs. For one thing, malaria infection will change household’s behaviors, such as patients might be inclined to be absence of indoor work which might possibly expose them to mosquitos’ biting. And people will also tend to stay in their own counties instead of going to work abroad, especially in the border areas. For another, malaria infection can lead to economic costs in the macro level, which cannot be assessed in this study at the household level. For example, with the nature of pandemic, malaria outbreaks can affect the willingness of international trade, tourism, and foreign investment, etc..

Another limitation of this study was the fact that mental stress and social costs of families with sick members were not included, which are in general very difficult to evaluate in a short period of time. Furthermore, the differences in epidemiology of malaria between regions and the absence of a generally accepted methodology to measure the economic impact of the disease made it difficult to compare the findings of this study directly with other studies.

Conducting qualitative research to obtain in-depth, locally relevant, and descriptive data on malaria costs should be encouraged. During the data analysis, we had limitations on capturing the correlations between the malaria costs with participants’ demographical, social, and economic status, e.g., gender, age, occupation, etc. Good-quality case management is crucial to efficiently use resources expended on the malaria elimination program: if case management is not done well, less is achieved and scarce health resources are wasted, which is likely to increase the risk of malaria transmission in the future.

## Conclusion

Malaria poses a significant economic burden on rural households and individuals in terms of direct and indirect costs. There is a need to develop effective strategies and interventions aimed at reducing malaria management costs at the household level in order to achieve the elimination goal in China. This study explored a systematic survey conducted in the main malaria endemic areas that endeavors to give an in-depth analysis of malaria management costs at the household level throughout the country. For effective management of imported malaria cases, surveillance and response systems should be carefully planned and well managed to ensure early diagnosis and prompt treatment. In addition, health education should be provided to all mobile laborers and other travelers before they travel abroad and after they return home. Health education materials should be provided to entry and exit border stations, and to local Centers for Disease Control and Prevention so that timely malaria tracking can be implemented. Training should also be provided to physicians to ensure the provision of accurate diagnosis and appropriate treatment.

## Availability of data and materials

In order to protect the privacy of surveyed malaria patients, we will not share the original copies of questionnaires. We would like to share statistical results of this study. If anyone needs these data, please contact the corresponding author for a soft copy.

## Additional file

Additional file 1: Multilingual abstracts in the six official working languages of the United Nations. (PDF 207 kb)

## Abbreviations

CNY: Chinese Yuan Renminbi
GDP: gross domestic product
*P. f*: *P. falciparum*
*P. v*: *P. vivax*
SD: standard deviation

## References

[1] Snow RW, Guerra CA, Noor AM, Myint HY, Hay SI (2005). The global distribution of clinical episodes of Plasmodium falciparum malaria. Nature.

[2] Murray CJ, Rosenfeld LC, Lim SS, Andrews KG, Foreman KJ, Haring D, Fullman N, Naghavi M, Lozano R, Lopez AD (2012). Global malaria mortality between 1980 and 2010: a systematic analysis. Lancet.

[3] Gallup JL, Sachs JD (2001). The economic burden of malaria. Am J Trop Med Hyg.

[4] Sachs J, Malaney P (2002). The economic and social burden of malaria. Nature.

[5] Teklehaimanot, Paola Mejia A (2008). Malaria and poverty. Ann N Y Acad Sci.

[6] McCarthy D, Wolf H, Bank W (1999). Malaria and growth. Policy research working paper.

[7] Bi Y, Tong S (2014). Poverty and malaria in the Yunnan province, China. Infect Dis Poverty.

[8] Bank W (1999). Economics of malaria: summary of facts and figures.

[9] Bank W (2001). Poverty reduction and the health sector.

[10] Worrall EBS, Hanson K (2005). Is malaria a disease of poverty? A review of the literature. Trop Med Int Health.

[11] Zhou X-N, Xia Z-G, Wang R-B, Qian Y-J, Zhou S-S, Utzinger J, Tanner M, Kramer R, Yang W-Z (2014). Feasibility and roadmap analysis for malaria elimination in China. Adv Parasitol.

[12] Yin JH, Yang MN, Zhou SS, Wang Y, Feng J, Xia ZG. Changing malaria transmission and implications in China towards National Malaria Elimination Programme between 2010 and 2012. PLoS One. 2013;8. http://journals.plos.org/plosone/article?id=10.1371/journal.pone.0074228. 10.1371/journal.pone.0074228 PMC3767829 24040210

[13] Feng J, Xia Z-G, Vong S, Yang W-Z, Zhou S-S, Xiao N (2013). Preparedness for malaria resurgence in china: case study on imported cases in 2000–2012. Adv Parasitol.

[14] Zhou SS, Wang Y, Li Y (2011). Malaria situation in the People’s Republic of China in 2010. Zhongguo Ji Sheng Chong Xue Yu Ji Sheng Chong Bing Za Zhi.

[15] Liu Y, Hsiang MS, Zhou H, Wang W, Cao Y, Gosling RD, Cao J, Gao Q (2014). Malaria in overseas labourers returning to China: an analysis of imported malaria in Jiangsu Province, 2001–2011. Malar J.

[16] Feng X-Y, Xia Z-G, Vong S, Yang W-Z, Zhou S-S (2014). Surveillance and response to drive the national malaria elimination program. Malar Control Elimination Program People’s Republic of China.

[17] Jackson S, Sleigh AC, Liu XL (2002). Cost of malaria control in China: Henan’s consolidation programme from community and government perspectives. Bull World Health Organ.

[18] Pei SJ, Ye JJ, Cheng F, Xu BZ, Jiang M, Webber RH (1998). Cost effectiveness of malaria case detection in low level malaria. China Public Health.

[19] Kidson C, Indaratna K (1998). Ecology, economics and political will: the vicissitudes of malaria strategies in Asia. Parassitologia.

[20] Tusting LS, Willey B, Lucas H, Thompson J, Kafy HT, Smith R, Lindsay SW (2013). Socioeconomic development as an intervention against malaria: a systematic review and meta-analysis. Lancet.

[21] Utzinger J, Tanner M (2013). Socioeconomic development to fight malaria, and beyond. Lancet.

[22] Sauerborn R, Shepard DS, Ettling MB, Brinkmann U, Nougtara A, Diesfeld HJ (1991). Estimating the direct and indirect economic costs of malaria in a rural district of Burkina Faso. Trop Med Parasitol.

[23] Jayawardene R (1993). Illness perception: social cost and coping-strategies of malaria cases. Soc Sci Med.

[24] Asenso-Okyere WK, Dzator JA (1997). Household cost of seeking malaria care. A retrospective study of two districts in Ghana. Soc Sci Med.

[25] Konradsen F, van der Hoek W, Amerasinghe PH, Amerasinghe FP (1997). Measuring the economic cost of malaria to households in Sri Lanka. Am J Trop Med Hyg.

[26] Attanayake N, Fox-Rushby J, Mills A (2000). Household costs of ‘malaria’ morbidity: a study in Matale district, Sri Lanka. Trop Med Int Health.

