# Supplementary material for: Economic cost analysis of malaria case management at the household level during the malaria elimination phase in The People’s Republic of China
Source: Infect Dis Poverty. 2016 Jun 3;5:50. doi: 10.1186/s40249-016-0141-x (PMC4891900; doi:10.1186/s40249-016-0141-x)
Supplement: Additional file 1: — Multilingual abstracts in the six official working languages of the United Nations. (PDF 207 kb) [file 40249_2016_141_MOESM1_ESM.pdf]

Translation of the abstract into the six official working languages of the United Nations

تحليل التكلفة الاقتصادية لتدبير حالات الملاريا على مستوى الأسر في الصين أثناء مرحلة القضاء على الملاريا

شانغ زيا، جن-زيانغ ما، ديو كوان وانغ، شي-زو لي، ديفيد رولنسن، شوي-سن زو، زياو-نونغ زو

موجز
الخلفية: في الصين، تشكل الملارياعبئا اقتصاديا كبيرا على الأسر. لتقييم العبء الاقتصادي من ناحية التكاليف المباشرة وغير المباشرة معنى من أجل تحسين فعالية برنامج القضاء على الملاريا في الصين.
الطرق: اختير عدد من مواقع الدراسة (ثمانية أقاليم في خمس مقاطعات) من المناطق الموبوءة بالملاريا في الصين، ممثلة المستويات المختلفة لمعدل حدوث، وتصنيف إمكانية الحدوث، والتطور الاقتصادي للملاريا. تم مسح عدة أسر توجد فيها حالات الملاريا (ع= 923) في الفترة الممتدة بين مايو وديسمبر 2012 لجمع معلومات عن العبء الاقتصادي للملاريا. استخدمت الاحصاءات الوصفية لوصف البرروفيل الأساسي لحالات الملاريا المختارة فيما يختص بالجنس، المجموعة العمرية، المهنة ونوع الملاريا. قُيِّمت التكلفة الاقتصادية للملاريا من حيث التكاليف المباشرة وغير المباشرة. وعُقدت مقارنات عن طريق استخدام اختبار خي مربع (أو اختبار **Z**) واختبار مان ويتني **U** بين حالات الملاريا مع الإشارة إلى مرضى الملاريا المحليين/الوافدين، مرضى المستشفيات/المرضى الخارجيين، ومستشفيات العلاج.
النتائج: كانت وسطي تكلفة الملاريا للحالة الواحدة 1691.23 يوان صيني (كانت التكلفة المباشرة 735.41 يوان صيني والتكلفة غير المباشرة 955.82 يوان صيني)، وهو ما يمثّل 11.1% من إجمالي دخل الأسرة. كان معدل تكلفة الحالة الواحدة للملاريا المحلية والوافدة 1087.58 يوان صيني و4,271.93 يوان صيني، على التوالي. وسطي تكلفة مريض الملاريا الذي تشخص حالته ويعالج في مشفى على مستوى الإقليم أو أعلى (3975.43 يوان صيني) تعادل 4.23 أضعاف تكلفة مريض الملاريا الذي تشخص حالته وتعالج على مستوى مستشفى القرية أوالبلدة (938.80 يوان صيني).
الاستنتاج: وجدت هذه الدراسة أن الملاريا دأبت على تشكيل عبء اقتصادي على الأسر من ناحية التكاليف المباشرة وغير المباشرة. هناك حاجة لتحسين فعالية المداخلات العلاجية لإنقاص تأثير تكلفة الملاريا، وخاصة بالنسبة لحلات العدوى الوافدة، من أجل القضاء على المرض في الصين.

Translated from English version into Arabic by Lina SM, through

TRANSLATORS WITHOUT BORDERS

中国疟疾消除阶段疟疾造成的家庭经济负担分析研究

夏尚，马金香，王多全，李石柱，David Rollinson，周水森，周晓农

摘要
引言：在中国疟疾给患者家庭带来了沉重的经济负担。通过有效评估因疟疾而带来的患者家庭直接和间接经济负担，可以更有效地实施中国疟疾消除计划。
方法：基于疟疾疫情、风险分级与社会经济发展水平，我们选择了5个省疟疾疫区中的8个乡镇作为研究地区。在2012年5月至12月间，针对923例疟疾感染病例开展疟疾家庭经济负担调查。我们使用描述统计学方法分析了这些病例的基本情况，包括他们的性别、年龄分布、职业以及感染疟疾类型。研究中将疟疾家庭经济负担分为直接负担和间接负担，通过卡方检验和曼惠特尼U检验的方法针对输入性或本地疟疾感染、医院治疗与否、以及在不同等级医院治疗等情况下开展疟疾感染家庭经济负担比较研究。
结果：研究中疟疾病例的平均家庭经济负担为1 691.23元（人民币），约占研究家庭年平均收入的11.1%，其中直接经济负担为735.41元，间接经济负担为955.82元。对于疟疾本地感染和输入性病例，平均经济负担分别为1 087.58元和4 271.93元。对于在县一级医院诊断和治疗的病例平均经济负担为3 975.43元，大约是在县镇一级医院诊断和治疗经济负担的4.23倍（938.80元）。
结论：本研究表明，疟疾感染给病患家庭带来了较大的经济负担。为了进一步推进在中国消除疟疾行动的进程，有效的降低疟疾病患家庭的经济负担，尤其是输入性疟疾病例，具有非常重要的意义。

Translated from English version into Chinese by Xia Shang

Analyse du coût économique de la gestion des cas de paludisme au niveau des ménages en Chine pendant la phase d'élimination du paludisme

Shang Xia, Jin-Xiang Ma, Duo-Quan Wang, Shi-Zhu Li, David Rollinson, Shui-Sen Zhou, Xiao-Nong Zhou

Résumé
Contexte: Le paludisme constitue un fardeau économique considérable pour les ménages chinois. Il est utile d'évaluer ce poids économique en termes de coûts directs et indirects à la fois, afin d'améliorer l'efficacité des programmes d'élimination du Paludisme en Chine.
Méthodes: Plusieurs sites d'étude (huit comtés dans cinq provinces) ont été sélectionnés dans l'aire d'endémie du paludisme en Chine, de façon à représenter les différents niveaux d'incidence de la maladie, de classification des

risques et de développement économique. Un certain nombre de foyers abritant des cas de paludisme (*n*=923) ont été relevés entre mai et décembre 2012 afin de recueillir des informations sur le poids économique du paludisme. Des statistiques descriptives ont été utilisées pour caractériser les profils de base des cas de paludisme sélectionnés en termes de sexe, de groupe d'âge, de profession et de type d'atteinte palustre. Le coût économique du paludisme a été évalué en termes de coût direct et indirect. Les comparaisons ont été réalisées à l'aide du teste de chi-deux (ou Z) et du test U de Mann-Whitney parmi les cas de paludisme, en faisant référence aux cas locaux/importés, aux patients hospitalisés/ambulatoires et aux hôpitaux dispensant le traitement.
Résultats: Le coût moyen du paludisme par cas était de 1691,23 CNY (coût direct 735,41 CNY et coût indirect 955,82 CNY), soit 11,1 % des revenus totaux d'un ménage. Le coût moyen par cas de paludisme local ou importé était respectivement de 1087,58 CNY et 4271,93 CNY. Le coût moyen d'un patient diagnostiqué et traité dans un hôpital de comté ou d'un échelon plus élevé (3975,43 CNY) était 4,23 fois plus élevé que celui du diagnostic et du traitement dans un hôpital rural ou de petite ville (938,80 CNY).
Conclusion: Notre étude a constaté que le paludisme imposait un fardeau économique significatif aux ménages, en termes de coûts directs aussi bien qu'indirects. Il est nécessaire d'améliorer les interventions afin de réduire cet impact économique du paludisme, et notamment des infections importées, et d'éradiquer la maladie en Chine.

Translated from English version into French by Suzanne Assenat, through

TRANSLATORS
WITHOUT BORDERS

Анализ экономических затрат для контроля малярии на уровне домохозяйств в Китае на этапе ликвидации малярии

Шанг Ся, Дзин-Сянг Ма, Дуо-Чуан Ванг, Ши-Джу Ли, Дэвид Роллинсон, Шуэй-Сен Джоу, Сяо-Нонг Джоу

Резюме
Подоплека: Малярия в Китае ложится тяжелым экономическим бременем на домохозяйства. Для оценки экономического бремени малярии в плане прямых и косвенных затрат важно повышать эффективность программы по ликвидации малярии в Китае.
Методы: Из эндемичных областей распространения малярии в Китае был выбран ряд исследовательских центров (восемь округов в пяти провинциях), представляющих разные уровни заболеваемости малярией, классификации риска, экономического развития. Ряд домохозяйств со случаями малярии (*n*=923) находился под наблюдением с мая по декабрь 2012 года с целью сбора информации об экономическом бремени малярии. Для описания основных профилей выбранных случаев малярии использовалась описательная статистика с учетом пола, возрастной группы, профессии и типа малярии. Экономические издержки малярии оценивались с точки зрения прямых и косвенных затрат. Сравнение случаев заболевания малярией проводилось с использованием теста хи-квадрат (или Z-критерия), а также критерия Уилкинсона-Манна-Уитни с указанием местных/завозных больных малярией, госпитализированных/амбулаторных больных и проходящих лечение в стационаре.
Результаты: Средняя сумма затрат составила 1 691.23 юаня на один случай малярии (прямые затраты составили 735.41 юаня и косвенные затраты 955.82 юаня), что составило 11.1% совокупного дохода домохозяйства. Среднестатистические издержки в случаях местной и завозной малярии составили 1 087.58 юаней и 4,271.93 юаней соответственно. Средние издержки в случае диагностирования и лечения малярии у больного на окружном уровне и выше (3 975.43 юаней) были в 4,23 раза выше, чем в деревенской или поселковой больнице или (938.80 юаней).
Заключение: Настоящее исследование показало, что малярия причиняет значительный экономический урон домохозяйствам – будь то прямые или косвенные издержки. Необходимо повышать эффективность мероприятий по снижению издержек, связанных с заболеваемостью малярией, особенно завозной инфекцией, с целью ликвидации этого заболевания на территории Китая.

Translated from English version into Russian by Jekaterina Merkuljeva, through

TRANSLATORS
WITHOUT BORDERS

Análisis del costo económico de la gestión de casos de malaria en el ámbito doméstico durante la fase de eliminación de la enfermedad en China.

Shang Xia, Jin-Xiang Ma, Duo-Quan Wang, Shi-Zhu Li, David Rollinson, Shui-Sen Zhou, Xiao-Nong Zhou

Resumen
Introducción: En China, la malaria representa una carga económica importante en el ámbito doméstico. El objetivo de evaluar esta carga económica en términos de costos directos e indirectos, se basa en mejorar la eficacia del programa de eliminación de la enfermedad en China.
Métodos: Se seleccionaron diversos sitios de estudio (ocho distritos pertenecientes a cinco provincias) en la zona donde la malaria es endémica en China, que representaran diferentes niveles de incidencia de la malaria, clasificación de riesgo y desarrollo económico. Se estudiaron varios hogares con casos de malaria *(n=923)* entre mayo y diciembre de 2012, con el fin de recolectar información sobre el costo económico de la enfermedad. Se utilizaron estadísticas descriptivas para delinear los perfiles básicos de determinados casos de malaria en relación con su género, grupo etario, ocupación y tipo de malaria. Se evaluaron los costos económicos de la malaria en función de los costos directos e indirectos. Se utilizó la prueba de ji-cuadrado (o prueba Z) y la prueba de U de Mann-Whitney para comparar casos de malaria en lo que refiere a pacientes locales/importados, pacientes hospitalizados/ambulatorios y hospitales de tratamiento.
Resultados: El costo promedio por caso de malaria resultó ser de 1 691,23 en moneda nacional (CNY) (el costo directo fue de 735,41 CNY y el indirecto, 955,82 CNY), lo que representa el 11,1% del total del ingreso familiar. El costo promedio por caso de malaria local e importada fue de 1 087,58 CNY y 4 271,93 respectivamente. El costo promedio por paciente de malaria al que se lo diagnosticó y trató en un hospital en el ámbito regional o superior (3 975,43 CNY) fue 4,23 veces mayor al costo por paciente de malaria al que se lo diagnosticó y trató en un pueblo o en un hospital municipal (938,80 CNY).
Conclusión: El estudió llegó a la conclusión de que la malaria representa una importante carga económica en el ámbito doméstico tanto en términos de costos directos como indirectos. Es imperativo mejorar la eficacia de la intervención para educir el impacto de los costos de la malaria, en especial de las infecciones importadas, a fin de eliminar la enfermedad de China.

Translated from English version into Spanish by Mpgorgone, through

TRANSLATORS
WITHOUT BORDERS
